# Detection of Microbial Agents in Oropharyngeal and Nasopharyngeal Samples of SARS-CoV-2 Patients

**DOI:** 10.3389/fmicb.2021.637202

**Published:** 2021-03-09

**Authors:** Tyler Seckar, Xiang Lin, Dipayan Bose, Zhi Wei, Joseph Rohrbaugh, Ronald G. Collman, Erle S. Robertson

**Affiliations:** ^1^Department of Otorhinolaryngology-Head and Neck Surgery, and Microbiology, The Tumor Virology Program, Abramson Comprehensive Cancer Center, Perelman School of Medicine, University of Pennsylvania, Philadelphia, PA, United States; ^2^Department of Computer Science, New Jersey Institute of Technology, Newark, NJ, United States; ^3^School of Medicine, University of Pennsylvania, Philadelphia, PA, United States

**Keywords:** PathoChIP, SARS-COV-2, COVID-19, microbes, respiratory agents, probes

## Abstract

The novel coronavirus outbreak started in December 2019 and rapidly spread around the globe, leading to a global pandemic. Here we reported the association of microbial agents identified in oropharyngeal and nasopharyngeal samples from patients with SARS-CoV-2 infection, using a Pan-microarray based technology referred to as PathoChIP. To validate the efficiency of PathoChIP, reference viral genomes obtained from BEI resource and 25 SARS-CoV-2 positive clinical samples were tested. This technology successfully detected femtogram levels of SARS-CoV-2 viral RNA, which demonstrated greater sensitivity and specificity than conventional diagnostic techniques. Simultaneously, a broad range of other microorganisms, including other viruses, bacteria, fungi and parasites can be detected in those samples. We identified 7 viral, 12 bacterial and 6 fungal agents common across all clinical samples suggesting an associated microbial signature in individuals who are infected with SARS-CoV-2. This technology is robust and has a flexible detection methodology that can be employed to detect the presence of all human respiratory pathogens in different sample preparations with precision. It will be important for differentiating the causative agents of respiratory illnesses, including SARS-CoV-2.

## Introduction

Viral pandemics that emerged over the last four decades were initiated across different geographic regions of the world and these includes the human immunodeficiency virus (HIV), severe acute respiratory syndrome (SARS) and Middle East respiratory syndrome (MERS) coronaviruses, influenza H1N1 virus, Ebola virus, Zika virus and most recently, the pandemic caused by SARS-CoV-2 which was initiated in Wuhan, China (Zhu et al., 2020). These viral pandemics are mainly due to zoonotic animal to human transmission, which then rapidly spread to the vulnerable human population. Importantly, the lack of a rapid, accessible and accurate molecular diagnostic technology deployment has restricted the rapid early response for detection of the virus, and thus the ability to control the outbreak.

The SARS-CoV-2 (the associated disease is known as COVID-19) was first reported in China in December 2019 (Ghinai et al., 2020), and it has spread to over 192 countries, and to date, infected more than 104 million people and close to 2.2 million deaths globally (World Health Organization, 2020). Infected individuals can shed this virus 2 days prior to symptom onset and thus person to person transmission rate is very high, the highest of all the recent viral pandemics (Gandhi et al., 2020). As a diagnostic tool, the RT-PCR approach is the most prevalent and widely accepted methods approved by all major health related regulatory bodies across the world (Tahamtan and Ardebili, 2020). Antigen tests have also become available, though it can only tell who was exposed to the virus and not those who are actively shedding. Its accuracy is also considerably lower than nucleic acid diagnostics (Guglielmi, 2020). The serological antibody test is another widely used technique employed for determination of exposure to the virus through the detection of antibodies against the viral antigens, most prominently the Spike and Nucleocapsid proteins (Lim and Lee, 2020), does not indicate active infection. However, all these techniques come with some limitations.

The conventional RT-PCR technique, although being the widely accepted technique for detection of SARS-CoV-2, has many potential drawbacks that can complicate the development of diagnostics including the many reports about false negatives or false positives (Wang et al., 2020; Yates et al., 2020). False negative outcomes are likely due to a variety of reasons, such as the quantity of viral RNA being below the limit of detection of the test, difficulty to amplify genomic regions due to sequence complexities, and loss of accurate annealing of primers to the template. False positive detection is most likely due to the fact that RT-PCR primers were annealing to other non-SARS-CoV-2 genome sequences that are capable of cross hybridizing to the primers of SARS-CoV-2 due to percent similarity to the other four Coronavirus family members associated with common cold. The serological tests are much faster compared to RT-PCR detection, but it takes several days to weeks following infection to develop an antibody response, depending on the individual [8], and does not indicate active infection. Moreover, the cross-reactivity between antibodies against SARS and SARS-COV-2, as well as other coronavirus family members, may also result in false positive detection (Lv et al., 2020). Therefore, for detection of initial infection, serological tests are not as effective and the possibility of false positives in these cases are much higher. There are other non-conventional techniques like the CRISPR–Cas12-based assay used for detection of SARS-CoV-2 from patient samples, referred to as the SARS-CoV-2 DNA Endonuclease-Targeted CRISPR Trans Reporter (DETECTR) (Broughton et al., 2020). This technique although very fast suffers from some drawbacks like off-target effects and tolerance to mismatches between the guide RNA and the target template (Jia et al., 2020; Teng et al., 2019).

We now report the development of a DNA microarray-based technology for detection of SARS-CoV-2 in patient samples. In previous studies we utilized this technology to report identification of a distinct microbiome in tumor samples from patients with different cancers, which we refer to as PathoChIP (Baldwin et al., 2014; Banerjee et al., 2018). We were also able to detect microbes that are rare and present in very low copy numbers. Therefore, this technology is highly sensitive and has a relatively quick turnaround time to obtain results based in our pipeline, which can give results within 24 hours after samples are received at the laboratory.

We have developed this technology as a potential resource that can be used as a sensitive diagnostic tool for early detection of highly infectious microorganisms in clinical samples, which includes all known respiratory viruses as well as SARS-CoV-2. DNA and RNA were isolated from nasopharyngeal (NP) and oropharyngeal swabs (OP), which were then subjected to whole transcriptome amplification (WTA) where the amplified products were used to hybridize against probes on specifically designed PathoChIPs containing sequences that are unique, conserved and frequently mutated across various regions of the SARS-CoV-2 genome. The current version of the PathoChip (V5) contains 60,000 probes and represents all known viruses, including SARS-CoV-2, and pathogenic microbes that comprise 250 helminths, 130 protozoa, 360 fungi, and 320 bacteria, totaling more than 6,000 accessions (Baldwin et al., 2014).

For SARS-CoV-2 detection, there are a total of 19 probes spanning the genome, consisting of 10 unique, 6 conserved, and 3 located in highly mutated regions, which are printed on the arrays. The arrey is also flexible in that new probes can be added and printed allowing for detection of new variants or new agents in a matter of days. Furthermore, the PathoChIP screening technology includes an amplification step that allows for detection of microorganisms and viruses that are present in low copy numbers or as fragmented genomes within samples. Different sample types can also be used, including swabs, tissues, cells, blood and other body fluids. Thus, PathoChIP allows a wide range of clinical samples, of various types, to be screened with high sensitivity and with relatively rapid detection of SARS-CoV-2 and other present microbial agents. Furthermore, as this technique relies on the hybridization of both microbial DNA and RNA, the sensitivity and the accuracy are far greater than the current conventional techniques employed. A more detailed description of the technology was previously described in multiple published studies (Baldwin et al., 2014; Banerjee et al., 2016).

## Results

### Detection of SARS-COV-2 Using the PathoChIP Array

**FIGURE 1:**
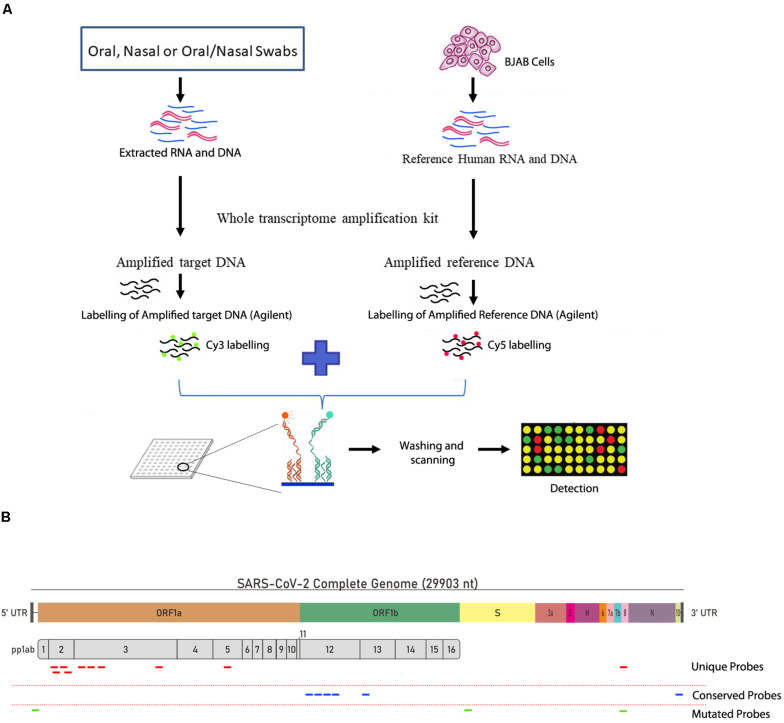
Workflow of the PathoChip technology and the design of the viral probes. **(A)** Schematic diagram showing the simplified workflow of the PathoChip technology starting from sample collection to data acquisition. **(B)** The structure of the SARS-COV-2 genome and the position of the different unique, conserved and mutated 60 nucleotide probes used to identify the SARS-COV-2 viral signatures.

**FIGURE 2:**
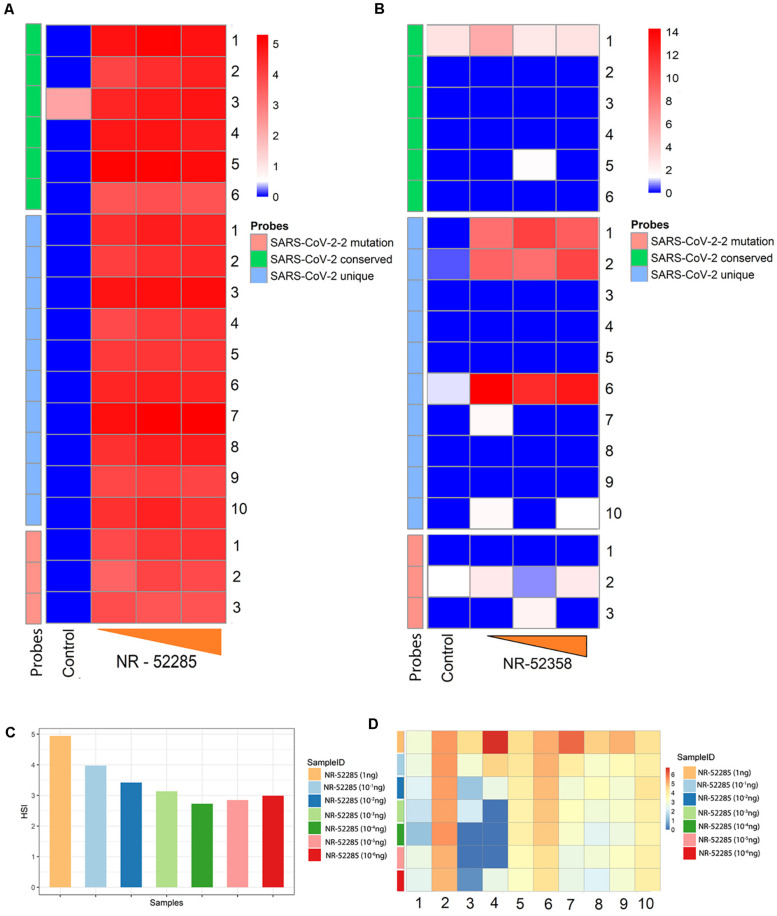
Determining efficacy and sensitivity of new conserved, mutated and unique SARS-CoV-2 probes using Agilent V5 microarrays. **(A)** Heatmap of hybridization signal intensity (HSI) for SARS-CoV-2 conserved, unique, and mutated probes positively detected reference sample NR-52285 with increasing hybridization input (1.5, 7.5, and 15 ng). **(B)** Heatmap of HSI for SARS-CoV-2 conserved, unique, and mutated probes positively detected from synthetic RNA NR-52358 containing fragments of the SARS-CoV-2 genome with increasing hybridization input (1, 5, and 10 ng). **(C)** Bar plot showing average HSI of all SARS-CoV-2 probes, including conserved, unique, and probes within the region susceptible to mutations, for positive control NR-52285 samples of decreasing hybridization inputs. **(D)** Heatmap of the HSI of individual SARS-CoV-2 unique probes for each sample of NR-52285 with increasing hybridization input (1-10^–6^ ng of RNA).

The SARS-CoV-2 probes, which include probes from conserved, unique and mutated regions, were able to successfuly detect the genomic RNA from the reference control sample (NR-52285) from SARS-related Coronavirus-2, obtained from BEI resources (Figure 1) which was established by National Institute of Allergy and Infectious Diseases (NIAID). The pipeline for collection, extraction, amplification, hybridization, and data extraction is shown using a schematic outline in Figure 1A. The location of all the 19 probes is shown on the SARS-CoV-2 genomic map (Figure 1B). Our initial test to determine the ability of these probes to perform in hybridization of SARS-CoV-2 genomic RNA used increasing concentrations (1.5, 7.5, and 15 ng) of reference sample NR-52285. The results (GEO submission accession number GSE166281) showed a strong hybridization signal intensity (HSI) across all six probes within the conserved regions, the three probes within the frequently mutated regions, and the ten probes within the unique regions of the genome and were nicely saturated across all probes present (Figure 2A). Furthermore, the HSI from genomic sample NR-52285 with an input of 1.5 ng had relatively similar hybridization for (approximately 5 HSI) all SARS-CoV-2 probes and was also maintained as the input RNA increased to 7.5 ng and 15 ng (Figure 2A). More specifically, the majority of conserved, unique and mutated probes show individual HSI’s, which were consistently measured at or above 4 for each increasing input of NR-52285 (Supplementary Figure 1). The results provide strong evidence demonstrating that all 19 probes were capable of detecting SARS-CoV-2 genomic RNA with high efficiency and intensities.

To determine the specificity of the probes, we used a sample NR-52358 (obatined from BEI resources), which is a synthetic RNA mixture containing fragments from the SARS-CoV-2 genomic regions encoding parts of ORF1ab, N and E. Our screen revealed that PathoChip consistently detected strong HSI for selected probes 1, 2 and 6 that lies within these unique regions of SARS-CoV-2 genome as expected (Figure 2B). As the hybridization input increased for NR-52358 from 5 ng to 10 ng, the HSI showed an increase for unique probes 1 and 2 but maintained an equally strong HSI for unique probe 6 (Figure 2B). However, conserved probe 1 and probe 2 which is against the highly mutable regions had very low levels of HSI. All other probes that did not show detectable hybridization was due to the absence of these specific regions of the viral genomic RNA in the mixture used from the reference sample (Figure 2B). This results clearly points out the specificity and sensitivity of the probes in differentiating the viral genome. To further confirm the specificity of the SARS-CoV-2 unique probes, we used 15 ng of RNA from canine corona virus UCD1 (NR-868) and recombinant murine corona virus (NR-43000) obtained from BEI resource for the hybridization as negative control. We observed that all probes had a HSI ≤ 0 after normalization (data not shown). These results confirm the specificity of the probes for SARS-CoV-2 used in PathoChIP.

### Determination of the Lower Limit of Detection for SARS-COV-2 Viral RNA Using PathoChip

To determine the lower limit of detection (LLOD) for the SARS-CoV-2 probes on PathoChIP, we serially diluted the positive control genomic RNA reference sample, NR-52285, to a dilution where the viral RNA copy number was less than 10. This was diluted with decreasing 10-fold concentrations of input to picograms levels (Table 1), which ranged from 7.12 × 10^7^ to 8 RNA molecules. The results showed a trend of consistently decreasing average HSI for all nineteen SARS-CoV-2 probes. The signals plateaued at an input of approximately 700 genomic RNA molecules (Figures 2C,D). Furthermore, an input of 700 RNA molecules (Table 1) measured a hybridization signal with an average intensity of approximately 2.75 (Figure 2C), and inputs of 80 and 8 genomic RNA molecules respectively, resulted in hybridizations with similar average HSI of SARS-CoV-2 probes when compared to hybridization of the input of 700 RNA molecules (Figure 2C and Table 1). This may be because at lower genomic RNA input, the stability and specificity of the probes hybridized to their region of the genomic RNA is significantly increased in regards to their intermolecular interactions. Overall, hybridization of input genomic RNA (NR-52285) demonstrated a consistent trend of decreasing HSI for SARS-CoV-2 probes, from 5 to 2.75 average HSI for all SARS-CoV-2 probes on dilution of input genomic RNA (Figures 2C,D).

**TABLE 1:** Calculations of different concentration of standard RNA sample.

Sample Name	Amount of WTA input of Sars Cov2 RNA (ng)	Amount of total ng in WTA product of 50 ul (ng)	Amount of WTA product used toward labeling (ug)	Portion of total WTA product that was used for labeling	Amount of SARS Co-2 RNA input that is represented in WTA used for labeling (ng)	Number of RNA molecules
NR-52285 1ng	1	8793.75	1	0.1137	1137.1 × 10^–4^	7.129 × 10^6^
NR-52285 0.1ng	10^–1^	8318.40	1	0.1202	120.2 × 10^–4^	7.536 × 10^5^
NR-52285 0.01ng	10^–2^	9355.30	1	0.1069	10.68 × 10^–4^	6.701 × 10^4^
NR-52285 0.001ng	10^–3^	8632.25	1	0.1158	1.15 × 10^–4^	7.262 × 10^3^
NR-52285 0.0001ng	10^–4^	8858.50	1	0.1129	0.112 × 10^–4^	7.077 × 10^2^
NR-52285 0.00001ng	10^–5^	7647.90	1	0.1308	0.0130 × 10^–4^	8.197 × 10^1^
NR-52285 0.000001ng	10^–6^	7247.10	1	0.1380	0.0013 × 10^–4^	8.650 × 10^0^
BJAB 20ng	N/A	7063.80	1	N/A	N/A	N/A

### Detection of SARS-CoV-2 in Patient Samples

**FIGURE 3:**
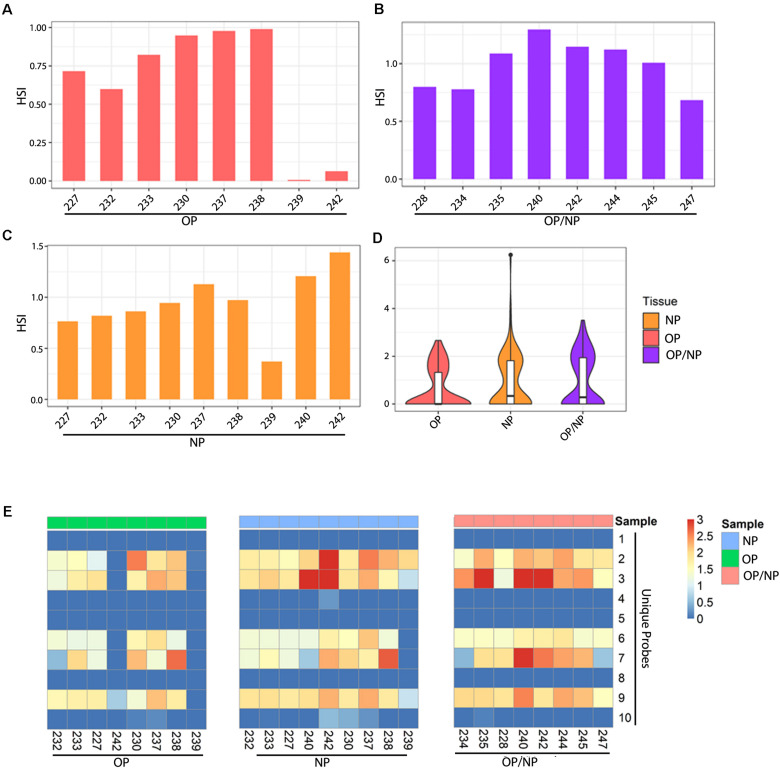
Patient swab samples screened for SARS-CoV-2 unique probes. Bar plots showing average HSI of unique SARS-CoV-2 probes for **(A)** Oropharyngeal (OP), **(B)** Nasopharyngeal (NP), and **(C)** Oropharyngeal/Nasopharyngeal (OP/NP) patient swab samples. **(D)** Violin plot depicting distribution of HSI for all unique SARS-CoV-2 probes for each patient swab sample type. **(E)** Heatmap showing HSI of SARS-CoV-2 unique probes for patient oral, nasal and oral/nasal swab samples.

A total of eight OP patient swab samples were screened, with six samples detecting an average HSI for unique SARS-CoV-2 probes consistently within a range of 0.50 and 1.00. Whereas two OP samples had significantly lower HSI, reported below 0.5 (Figure 3A). All eight combined samples of OP/NP patient swabs reported a consistently strong signal intensity, which ranged from approximately from 0.75 to 1.25 (Figure 3B). For the nine patient samples obtained from NP swabs, five were found with consistently strong signal intensity ranging from 0.75 to 1.00 (Figure 3C). Three NP swabs from patients were reported with an average HSI greater than 1.00, and one NP swab from patients had an average HSI below 0.50 (Figure 3C). Overall, the combined samples of OP/NP swabs showed an even distribution of HSI for unique SARS-CoV-2 probes when compared to samples obtained from only NP or OP swabs (Figure 3D). The OP patient swabs showed a tendency for lower HSI to the unique SARS-CoV-2 probes, and samples from NP patient swabs showed a similar distribution pattern of HSI to that of the combined OP and NP samples, as well as the OP samples, with some outliers as shown on the violin plots (Figure 3D).

To further investigate the activity of the ten unique SARS-CoV-2 probes, we analyzed their hybridization across the samples. Our data showed that five unique probes were consistently detected among all of the patient swab samples, specifically the unique probes 2, 3, 6, 7, and 9 (Figure 3E). Of the eight OP patient swab samples, six samples showed positive detection by these five select unique probes (Figure 3E left panel, Figure 3A). OP patient swab sample 242 only showed positive detection with unique probe 9 at a much lower HSI, whereas sample 239 did not show any positive detection for any of the unique SARS-CoV-2 probes (Figure 3A). However, OP patient swab samples 230 and 238 reported a significantly strong HSI which measured at approximately 2.5, for both unique probes 2 and 7 (Figure 3E).

The eight combined OP/NP patient swab samples all consistently showed hybridization to the select unique probes 2, 3, 6, 7, and 9 (Figure 3E right panel). Patient swab samples 234 and 247 had significantly lower HSI for unique probe 7, as compared to the remaining OP/NP patient swab samples (Figure 3E right panel). However, the combined OP and NP patient swab sample 240 reported a significantly strong hybridization signal intensity for both unique probes 3 and 7 (Figure 3E right panel). Additionally, patient samples 235 and 242 showed a significantly higher HSI for unique probe 3 (Figure 3B).

**FIGURE 4:**
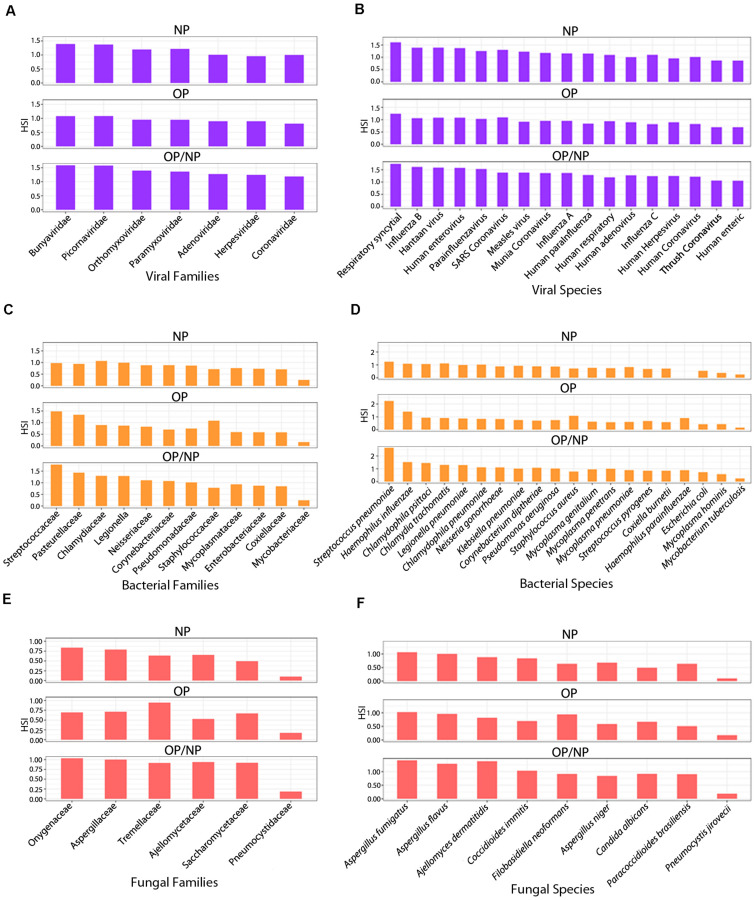
Respiratory agent signature families identified in patient swab samples. Series of bar plots representing average HSI of signature **(A)** viral, **(C)** bacterial and **(E)** fungal families and **(B)** viral, **(D)** bacterial and **(F)** fungal species identified in Oropharyngeal (OP), Nasopharyngeal (NP), and Oropharyngeal/Nasopharyngeal (OP/NP) patient swab samples.

Of the nine NP patient swab samples, eight showed a consistent HSI for the selected unique probes 2, 3, 6, 7, and 9 (Figure 3E middle panel). Specifically, NP patient swab sample 239 only had positive signal for unique probes 2, 3, and 9 (Figure 4E middle panel). Notably, three NP patient samples showed significantly high HSI for certain unique probes, specifically sample 240 which showed positive signals for unique probe 3, sample 242 showed positive signal for unique probes 2 and 3, and sample 238 showed positive signal for unique probe 7 (Figure 3E).

### Families and Species of Other Respiratory Viruses Identified in Patient Swab Samples

Seven signature viral families were strongly detected amongst the swab samples from COVID patients and were consistently identified in all swab sample types, including NP, OP and combined OP/NP (Figure 4A). Our analyses were to determine which organisms were commonly seen across the different sample types which may provide some insights into the potential virome and microbiome that are symbionts or commensals in the nasopharyngeal and oropharyngeal niche that may contribute to disease severity or a level of protection from pathogenesis in infected patients.

Among the NP samples from patient swabs, the respiratory agents from the viral families Bunyaviridae, Picornaviridae and Paramyxoviridae reported the strongest HSI, measuring greater than 1.0 (Figure 4A). Coronaviridae reported the second lowest HSI among the other signatures of families of respiratory agents that were clearly detected, measuring at approximately 0.8 HSI (Figure 4A). Amongst the OP swab samples from COVID patients, signatures of all seven viral families associated with respiratory infections had consistently similar HSI, ranging between 0.5 and 0.8 signal intensity (Figure 4A). Specifically, viral families belonging to the Bunyaviridae and Picornaviridae had the highest HSI levels reported for the samples from patient OP swabs at approximately 0.8 HSI (Figure 4A). Coronaviridae was also reported with an HSI of approximately 0.9, when compared to the six other respiratory viral families detected (Figure 4A). Overall, all seven respiratory viral families had similar HSI for all swabs of patient samples (Figure 4A). Bunyaviridae and Picornaviridae family members were the most predominant respiratory viral agents (Figure 4A). The combined OP/NP swabs of patient samples reported the strongest HSI across all seven respiratory viral families identified, including Coronaviridae, when they were compared to the NP and OP patient swab samples (Figure 4A).

On further analyses we identified, a total of 17 signatures of viral respiratory agents that were identified among patient samples and they all were detected from each swab sample type obtained (Figure 4B). Among the NP patient samples, all viral signatures had consistent high signal intensities (Figure 4B). Signatures for respiratory syncytial virus, Influenza B, Hantaan virus, and human enterovirus reported the highest signal as determined by HSI of 1.25 or greater (Figure 4B). The Human enteric virus reported the weakest HSI among the seventeen total signature viral agents identified, measuring approximately 0.80 HSI (Figure 4B). Notably, human coronavirus was detected at a signal of approximately 1.0 HSI (Figure 4B). The seventeen respiratory viral agents were detected among the OP patient swab samples and maintained consistent in signal intensity, ranging from 0.75 to 1.25 HSI (Figure 4B). These results were very similar to what was seen in the NP samples and the combined OP and NP samples screened. Specifically, the respiratory syncytial virus (RSV) had the highest HSI signal reported for the OP patient samples, measuring approximately 1.25 HSI (Figure 4B). Whereas human enteric virus (HEV) reported the lowest HSI as compared to the other signatures identified from other respiratory viral agents detected (Figure 4B). Specifically, human coronavirus was detected at signal levels between HSI of that seen for RSV and HEV (Figure 4B). The signal for RSV was the predominant viral respiratory agent detected amongst the patient samples (Figure 4B). Furthermore, among the three different patient sample types, HEV was consistently the respiratory viral agent with the weakest signal when compared to the signals for the other signature viruses identified (Figure 4B). The combined OP/NP samples and the NP samples show a much higher range of HSI distribution among viral agents identified, and further reporting an overall stronger HSI for all seventeen respiratory viral agents, including human coronaviruses, when compared to OP sample types.

### Families and Species of Other Respiratory Bacteria Identified in Patient Swab Samples

We then investigated the bacterial families that were positively detected in the patient swab samples. Our goal were to identify the bacterial families that were similar across the different samples. Twelve families were consistently identified in each of the patient sample types (Figure 4C). Among the NP swab samples, eleven out of the twelve bacterial family signatures showed consistently similar HSI ranging between 0.6 and 0.9 (Figure 4C). Respiratory bacterial agents in the families Chlamydiaceae, Legionellaceae, and Steptococcaceae had the strongest HSI, which were greater than 0.8 (Figure 4C). The Mycobacteriaceae family reported the lowest HSI as compared to signatures of the other respiratory bacterial agents detected, which measured a signal intensity at approximately 2.5 (Figure 4C). Amongst the OP samples from patient swabs, signatures from the twelve respiratory bacterial families were positively detected but were heterogeneous in terms of signal intensity (Figure 4C). Specifically, signatures for the Streptococcaceae, Pasteurellaceae and Staphylococcaceae families had the strongest HSI reported for the OP samples from patient swabs, measuring an HSI of approximately 8 or higher (Figure 4C), whereas signatures for the Mycobacteriaceae family showed the lowest HSI when compared to signatures from other families of respiratory bacterial agents detected (Figure 4C). Our investigation of samples that were combined OP and NP swabs showed that signatures for all twelve families of respiratory bacterial agents were positively identified. Signatures from families of Streptococcaceae and Pasteurellaceae reported the strongest signal intensity of the total signatures, each measuring an HSI greater than 1.0 (Figure 4C). The Mycobacteriaceae family had the lowest HSI as compared to signatures of bacterial families from other respiratory agents detected, with a HSI of approximately 0.25 (Figure 4C).

Reviewing all the data suggests that the Steptococcaceae and Pasteurellaceae families appear to be the predominant respiratory bacterial families identified amongst the different types of samples (Figure 4C). Furthermore, among all the patient sample types, the signatures within the Mycobacteriaceae family consistently had the least hybridization signal when compared to signatures from other bacterial families identified (Figure 4C). The NP patient samples showed a more consistent HSI among the signatures for the bacterial families identified, whereas the OP patient samples were more inconsistent in terms of HSI distribution (Figure 4C). However, the combined OP/NP patient samples were similar in HSI distribution across the bacterial families identified, but overall report a greater or similar HSI for the twelve respiratory bacterial agents when compared to NP patient samples (Figure 4C).

Twenty signatures for specific respiratory bacterial agents were identified within the three patient sample types collected (Figure 4D). Among the NP patient swab samples, only 19 out of the 20 respiratory bacterial agents were detected. Specifically, *Haemophilus parainfluenzae* was not identified in the NP samples (Figure 4D). Of the nineteen respiratory bacterial signatures identified, all had a similar and consistent signal distribution of HSI between 0.25 and 1.1 (Figure 4D). *Streptococcous pneumoniae* was identified with the strongest signal intensity compared to the remaining bacterial signatures detected (Figure 4D). *Escherichia coli*, *Mycoplasma hominis* and *Mycobacterium tuberculosis* were reported as having the weakest HSI, measuring 0.5 or lower (Figure 4D). All twenty respiratory bacterial agents were detected among the OP patient swab samples, however the distribution of HSI appeared to be variable (Figure 4D). Specifically, bacterial agent *Streptococcous pneumoniae*, *Haemophilus influenzae*, and *Staphylococcus aureus* had the strongest HSI reported for the OP patient samples (Figure 4D), and *Mycobacterium tuberculosis* showed the lowest HSI, at approximately 0.1, as compared to the signals for the other respiratory bacterial agents identified (Figure 4D). All twenty respiratory bacterial agents were detected in the combined OP/NP patient samples, where *Streptococcous pneumoniae* maintained the strongest signal intensity, measuring 2.5 HSI (Figure 4D). Furthermore, both *Haemophilus influenzae* and *Chlamydophila psittaci* showed, similarly, strong signal intensities, reported at 1.50 HSI (Figure 4D). As expected, *Mycobacterium tuberculosis* reported the weakest signal intensity (Figure 4D). The signatures for the remaining sixteen bacterial species detected among combined OP/NP samples maintained a similar and consistent HSI distribution, ranging between 0.5 to 1.25 HSI (Figure 4D).

Overall, *Streptococcous pneumoniae* appeared to be the predominant bacterial respiratory agent that was detected amongst all the patient samples (Figure 4D). NP patient swabs showed the most consistent distribution of HSI for each respiratory bacterial agent positively detected, albeit generally low in signal intensity throughout, whereas OP patient swabs had a more variable pattern of HSI among the bacterial signatures (Figure 4D). Specifically, OP/NP patient swab samples show an overall stronger HSI for the predominant bacterial agents, *Streptococcus pneumoniae* and *Haemophilus influenzae*, but maintained a consistently higher HSI among the remaining bacterium, when compared to OP and NP patient swabs (Figure 4D).

### Families and Species of Other Respiratory Fungus Identified in Patient Swab Samples

We also investigated the fungal agents that were commonly associated with the collected sample types. Signatures representing six respiratory fungal families were detected in the patient samples, all consistently identified in NP, OP, and OP/NP sample types (Figure 4E). Of the NP patient samples, five out of the six signatures of fungal families showed a consistently similar signal intensity, ranging between 4 and 7.5 HSI (Figure 4E). Respiratory fungal families Onygenaceae and Aspergillaceae were reported to have the strongest HSI as compared to signatures from the other agents identified with signals greater than 0.7 HSI (Figure 4E). Pneumocystidaceae reported the lowest signal intensity when compared to the other signatures from other respiratory fungal agents (Figure 4E). The six families of respiratory fungal agents were detected among all of the OP patient samples but varied widely in signal intensity (Figure 4E). More specifically, Tremellaceae had the strongest signal reported for the OP patient samples and the Pneumocystidaceae family reported the lowest signals when compared to signatures of the other respiratory fungal families identified (Figure 4E). Five out of the six total respiratory fungal families showed consistently strong HSI signals among the OP/NP patient samples, ranging between 0.875 and 1.0 HSI (Figure 4E). The families Onygenaceae and Aspergillaceae maintained the strongest signal intensity of all signatures from the families detected, each measuring an HSI of 1.0 (Figure 4E). As expected, Pneumocystidaceae showed the lowest HSI signal as compared to signatures from the other respiratory fungal families detected (Figure 4E). The Onygenaceae and Aspergillaceae families were the predominant respiratory fungal families detected amongst the patient samples (Figure 4E). Furthermore, among all the sample types screened, Pneumocystidaceae family was the weakest respiratory fungal signal when compared to the other signatures from the fungal families identified (Figure 4E). Notably, between NP and OP patient samples, the NP samples showed a more consistent signal among signatures of fungal families identified, whereas OP patient samples were more widely varied in signal across the six families identified (Figure 4E). The combined OP/NP patient samples showed similar HSI distribution among the signatures of the families identified and demonstrated an overall greater HSI for the six respiratory fungal families when compared to NP and OP patient samples (Figure 4E).

A total of nine signature respiratory fungal agents were identified among all of the patient samples, with each consistently identified among each patient swab type (Figure 4F). Of the NP patient samples, both *Aspergillus fumigatus* and *Aspergillus flavus* maintained the strongest signal intensities (Figure 4F). Whereas *Pneumocystis jirovecii* reported the weakest signal intensity at approximately 0.1 HSI (Figure 4F). Of the remaining seven fungal signatures identified, they maintained consistent signal intensities across the NP swab samples (Figure 4F). All nine respiratory fungal agents were positively detected among the OP patient samples, where *Aspergillus fumigatus*, *Filobasidiella neoformans (Cryptococcus neoformans)* and *Aspergillus flavus* has the strongest signal intensities (Figure 4F) while *Pneumocystis jirovecii* the weakest signal intensity of all the signature fungal species identified (Figure 4F). *Candida albicans* were also detected in all the samples with intermediate signal intensities among all the other fungal species. Signatures for the remaining five fungal species maintained similar and consistent distribution of signal intensities (Figure 4F). The combined OP/NP patient samples, *Aspergillus fumigatus, Aspergillus flavus* and *Ajellomyces dermatitidis (Blastomyces dermatitidis)* maintained the strongest signal intensities (Figure 4F). As above, *Pneumocystis jirovecii* reported the weakest signal intensity (Figure 4F). The remaining five fungal respiratory species maintained a strong and consistent HSI distribution (Figure 4F).

Overall, *Aspergillus fumigatus* appears to be the predominant fungal respiratory agent detected amongst all of the patient samples (Figure 4F). Furthermore, among all patient sample types, *Pneumocystis jirovecii* was consistently the fungal respiratory agent with the weakest HSI in all fungal signatures identified (Figure 4F). All patient sample types had a varied distribution of HSI for the identified respiratory fungal agents. However OP/NP patient swabs reported a consistently similar and stronger profile of HSI for signatures detected than NP and OP patient swabs (Figure 4F).

## Discussion

The high efficiency of the metagenomic multiplex assay to identify non-human, microbial RNA and DNA genomes is a hallmark of the PathoChIP technology. In the current pandemic, identifying the viral infection during the early stage of the disease is crucial for curbing the spread of the disease. Limitations of some conventional detection techniques lies in the sensitivity due to low copy numbers of the virus at the initial stage of the infection, which has slowed efforts to diagnose patients positive for SARS-COV-2 during this early period after infection. Thus, a more sensitive test will increase diagnostic power and allow physicians to quickly identify and isolate patients that are positive and ultimately minimize the potential spreading, therefore containing transmission and the R-number. The current version of the PathoChip (V5) that was designed specifically to detect the SARS-CoV-2 virus, containing probes in 10 unique, 6 conserved and 3 within the regions frequently mutated. The probes are 60 nucleotides long and were designed in a way to detect different regions of the SARS-CoV-2 genome. Of the 10 unique probes, nine were designed for different regions of ORF1ab and one probe was designed for ORF8. The NSP12 (RDRP) genomic region is one of the most conserved within the coronavirus family, and so we designed three out of the six conserved probes to target this particular region.

A number of reports have shown that the virus is gaining mutations that are located in specific regions of the viral genome. Based on the available mutated genomic sequences, we also designed 3 probes to detect sequences that are from these regions frequently mutates on the virus. This version of PathoChIP was also able to detect all other respiratory microorganisms that were common to the different types of samples that also contained SARS-CoV-2.

We have used three different concentrations of the reference genome (NR-52285) to hybridize to probes in the unique, conserved and the mutated regions of SARS-CoV-2 on the PathoChip. All 19 probes successfully detected the SARS-CoV-2 reference genome (Figure 2A). To determine the specificity of our assay we used another reference sample (NR-52358) which contains fragments from genomic region of ORF1ab, N and E of SARS-CoV-2. The results clearly showed that our probes only detected the samples that have genomic fragments that were complementary to the probe sequences and showed no detectable signal for the probes whose complementary viral RNA was not present in the reference sample. To further confirm the sensitivity of the technology, we used different concentrations, ranging from 10^6^ to less than 10 reference viral RNA copies (NR-52285). We successfully detected the presence of the viral RNA genomes with precision even at dilutions of less than 10 copies. This level of specificity and sensitivity demonstrates that the PathoChip technology is a significant improvement compared to other diagnostic technologies available.

To further validate the effectiveness of the PathoChip for detecting SARS-CoV-2 in patient samples, we have screened NP, OP and combined NP and OP swabs, and following the same pipeline was able to detect the SARS-CoV-2 viral nucleic acids in samples from CoVID-19 patients. These results clearly demonstrated that the PathoChip technology is effective and accurate in detecting the SARS-CoV-2 nucleic acids.

There are some preliminary reports about the co-infection of different type of viruses (Kim et al., 2020) and bacteria (Lansbury et al., 2020) that have been detected in the swab samples of the SARS-CoV-2 infected patients. The present study is designed to get a comprehensive idea of the total microbiome present in the nasopharyngeal and the oropharyngeal swabs of SARS-CoV-2 patients. This version of the PathoChIP also contains probes for other respiratory microorganisms. Therefore, we further analyzed the patient samples to determine the presence of other respiratory infectious agents that may provide clues as to the virome and microbiome signatures that are associated with SARS-CoV-2 in CoVID-19 patients. We detected 7 viral, 12 bacterial and 6 fungal families that were common in the samples screened. This included 17, 20 and 11 respiratory viral, bacterial and fungal signature agents, respectively. Respiratory syncytial virus was the most predominant viral respiratory agent, while the human enteric virus was consistently the least predominant respiratory viral agent detected amongst all the patient samples. SARS-CoV-2 was also detected, as expected, but interestingly was not the most dominant viral agent present (Figure 4B). The detection of other viral agents may also be due to similarities of probe sequences to other family members that have not been previously identified in that viral family. *Streptococcous pneumoniae* and *Mycobacterium tuberculosis* signature probes were the most and the least predominant microorganisms, respectively, that were identified in all of the patient samples. *Streptococcous pneumoniae* and *Haemophilus influenza* were two other bacterial species that maintained a high hybridization signal in the majority of samples. Similarly, *Aspergillus fumigatus* and *Pneumocystis jirovecii* are the most and the least abundant fungal species, respectively, detected all across the patient samples.

The PathoChIP technology provides a comprehensive tool to detect pathogenic microorganisms, including SARS-COV-2 that may be present in very low copy numbers from different types of patient samples, while also providing a detailed overview about the presence of other co-infectious agents. The presence of other bacterial and fungal respiratory agents that are common in these patients may provide additional paracrine activities that can trigger inflammatory responses and lead to differences in severity of disease associated with SARS-CoV-2 infection. Notably, the PathoChIP can detect less than 10 copies of the RNA genome for SARS-CoV-2 in a complex background of human genomic DNA, which makes it a technology with potential utility for surveillance in populations or communities where information regarding asymptomatic carriers will be important to identify and tracethus minimizing the spread of this highly transmissible agent.

## Materials and Methods

### Probe Selection, Validation and Sensitivity Testing of SARS-CoV-2 Probes

Probes for SARS-CoV-2 were selected by choosing regions of unique and conserved genomic sequences across the approximately 30,000 nucleotides which make up the entire published sequence of the virus (Wu et al., 2020). These selected regions were then analyzed for 60 nts that do not cross hybridize to any other known sequences in the databases for human, mouse, yeast and plants. They were also determined to have no cross reactivity against other sequences in the GenBank, as well as within the PathoChIP sequence database. The PathoChIP consists of 60 synthetic chromosomes containing greater than 6000 accessions (Baldwin et al., 2014) suggesting that the probes in the microarrays are designed based on more than 6000 different microorganisms including virus, bacteria, fungi and protozoans. The unique and conserved probes for SARS-CoV-2 were designed from the NCBI reference genome NC_045512.2, and probes for detection of mutated regions were designed from MT049951.1 (Wu et al., 2020). The whole PathoChIP design is deposited in GEO submission (GSE166281).

Samples for use as positive controls for SARS-CoV-2 were obtained from BEI Resources and are detailed in Table 1. The quality of each positive control sample was determined by A260/280 measurements and prepared for Whole Transcriptome Amplification (WTA) using the TransPlex Complete Whole Transcriptome Amplification Kit (Sigma-Aldrich, St. Louis, MO). For each positive control sample, 100 ng of RNA was used as input for WTA. Reference human DNA and RNA was extracted from the human B cell line, BJAB (obtained from ATCC), to serve as a reference for determining cross-hybridization of probes to human sample amplified genome. After purifying the WTA products (PCR purification kit, Qiagen, Germantown, MD, United States), the quality was determined using A260/280 measurements and 1μg of the amplified products from the positive control specimens were labeled with Cy3 and the human reference was labeled with Cy5 (SureTag labeling kit, Agilent Technologies, Santa Clara, CA, United States). All labeled specimens were purified and hybridized to the PathoChIP microarrays, as previously described (Baldwin et al., 2014; Banerjee et al., 2015). For each PathoChIP array, the Cy3 labeled specimen and Cy5 labeled reference were hybridized together and maintained at constant rotation at 65°C for approximately 12 h. The slides were then washed and scanned for visualization using an Agilent SureScan G4900DA array scanner.

### Sample Preparation and Microarray Processing

Following informed consent under protocols approved by the University of Pennsylvania IRB (protocol #823392), clinical specimens were obtained from 15 patients admitted to the hospital with documented COVID19 (Table 2). Samples were obtained using flocked swabs (Copan Diagnostics, Murrieta, CA, United States), eluted in viral transport media (VTM), then heat inactivated at 56°C for 30 minutes prior to transfer to the laboratory. Specimens included 8 paired oropharyngeal (OP), 9 nasopharyngeal (NP) swabs, and 8 combined oropharyngeal/nasopharyngeal OP/NP. Two blank swabs resuspended in VTM media were also prepared from the same environment where the clinical samples were prepared in VTM to be used as controls. Both patient samples and blank controls were micro-centrifuged at 4°C at 15,000 rpms for 20 min which will collect all cells, virions and other microbial agents eluted in the VTM. The pelleted samples were then resuspended for simultaneous DNA and RNA extraction (AllPrep DNA/RNA FFPE Kit, Qiagen, Hilden, Germany). The quality of extracted nucleic acids was determined by A260/280 measurements. WTA were performed using the TransPlex Complete Whole Transcriptome Amplification Kit (Sigma-Aldrich, St. Louis, MO, United States) for each specimen using extracted DNA and RNA, as described previously (Baldwin et al., 2014; Banerjee et al., 2016; Banerjee et al., 2018). Reference control human DNA and RNA was extracted from the human B cell line, BJAB (obtained from ATCC, Manassas, VA, United States). After purifying the WTA products (PCR purification kit, Qiagen, Germantown, MD, United States), their quality was checked using A260/280 measurements and 1μg of the amplified products from the patient and reference genomes were labeled separately with Cy3 and Cy5, respectively (SureTag labeling kit, Agilent Technologies, Santa Clara, CA, United States). The detailed calculations for amounts of RNA and number of SARS-CoV-2 molecules spiked in samples in a background of BJAB DNA/RNA are provided in Table 1. All labeled controls and experimental specimens were purified and hybridized to the PathoChIP microarrays, as previously described (Baldwin et al., 2014; Banerjee et al., 2015). For each PathoChIP array, a Cy3 labeled specimen and a Cy5 labeled reference were combined and hybridized together in constant rotation at 65°C for 12 h. The slides were then washed and scanned for visualization using an Agilent SureScan G4900DA array scanner.

**TABLE 2:** Patient information.

Patient ID	Sample(s)	Days post Sx Onset	Age	Gender	Race	Hispanic / Latin	COVID clinical test
227	NP VTM; OP VTM	10	54	M	Black	No	Xpert^®^Xpre - Cepheid GeneXpert System
228	NP/OP VTM	12	65	F	other	No	Xpert^®^Xpre - Cepheid GeneXpert System
230	NP VTM; OP VTM	11	55	M	Black	No	Quest Diagnostics RT-PCR
232	NP VTM; OP VTM	7	54	M	Black	No	Quest Diagnostics RT-PCR
233	NP VTM; OP VTM	17	73	F	Black	No	Xpert^®^Xpre - Cepheid GeneXpert System
234	NP/OP VTM	9	75	M	Black	No	Xpert^®^Xpre - Cepheid GeneXpert System
235	NP/OP VTM	2	76	F	Black	No	Xpert^®^Xpre - Cepheid GeneXpert System
237	NP VTM; OP VTM	9	64	F	Black	No	Xpert^®^Xpre - Cepheid GeneXpert System
238	NP VTM; OP VTM	9	57	M	Black	No	Cobas SARS-CoV-2 assay (Roche Molecular Systems
239	NP VTM; OP VTM	13	73	M	Black	No	Xpert^®^Xpre - Cepheid GeneXpert System
240	NP VTM	10	85	F	Black	No	Xpert^®^Xpre - Cepheid GeneXpert System
242	NP VTM; OP VTM	3	58	F	Black	No	Xpert^®^Xpre - Cepheid GeneXpert System
244	NP/OP VTM	24	72	F	other	No	Xpert^®^Xpre - Cepheid GeneXpert System
245	NP/OP VTM	11	50	M	Black	No	Xpert^®^Xpre - Cepheid GeneXpert System
247	NP/OP VTM	11	60	F	White	No	ePlex - SARS-CoV-2 assay (GenMark Diagnostics

### Data Extraction, Normalization and Analysis

PathoChIP is a two-channel array, with one green channel for profiling sample of interest and one red channel for reference genomic DNA (from human B cell line). Agilent Feature Extraction software was used for extraction of the raw green and red signal data from the microarray images captured by the G400DA scanner. There are spiked-in human intergenic reference probes (HRP) that were used for raw data normalization. A scale factor, which was inferred from HRP, was set as 0.3 to control the effect from red to green signal. Specifically, we multiply the log2 red signal by the scale factor to get the normalized background signal which was then subtracted from the log2 green signal to get the final normalized data. The normalized value of a probe is called hybridization signal intensity (HSI) which indicates the abundance of the species of the probe in the tested sample. All negative HSI (< 0) were set to 0 which indicates negligible signal or the absence of the species in the tested sample. HSI 0-1 means the signal intensity of a probe is 1X to 2X to the background signal. This is a positive but weak signal and indicates the low abundance of the species in the tested sample. HSI > 1 means the signal intensity of a probe is higher than 2X of the background signal. This is a positive and strong signal and indicates the high abundance of the microbial species in the tested sample. The HSI of the probes identified for specific organisms were used to create the bar plots, heatmaps and violin plots in this study. The signals from the blank swabs control arrays were used to subtract from all other arrays so that we would eliminate any potential background signals that were due to the blank swabs, VTM or other microbes that were captured in the transfer. All analyses were focused on identifying the SARS-CoV-2 probes and the common signals across the 3 samples types (OP, NP, OP/NP collected).

## Data Availability Statement

The data presented in the study are deposited in the GEO repository, accession number GSE166281.

## Ethics Statement

The studies involving human participants were reviewed and approved by University of Pennsylvania IRB (protocol #823392). The patients/participants provided their written informed consent to participate in this study.

## Author Contributions

TS, DB, and JR performed the experiments and collected the data. XL and ZW analyzed the data. DB drafted the manuscript. RC, TS, and ER revised and edited the manuscript. RC helped with the clinical samples. ER conceptualized the project. All authors contributed to the article and approved the submitted version.

## Conflict of Interest

The authors declare that the research was conducted in the absence of any commercial or financial relationships that could be construed as a potential conflict of interest.
