# Supplementary material for: Detection of Microbial Agents in Oropharyngeal and Nasopharyngeal Samples of SARS-CoV-2 Patients
Source: Front Microbiol. 2021 Mar 9;12:637202. doi: 10.3389/fmicb.2021.637202 (PMC8006406; doi:10.3389/fmicb.2021.637202)

**Supplementary Figure Legends**

**Supplementary Figure 1:** HSI of each of Sars-CoV-2 conserved, unique and mutated probes for sample NR-52285 with increasing hybridization input (1.5, 7.5 and 15 ng).


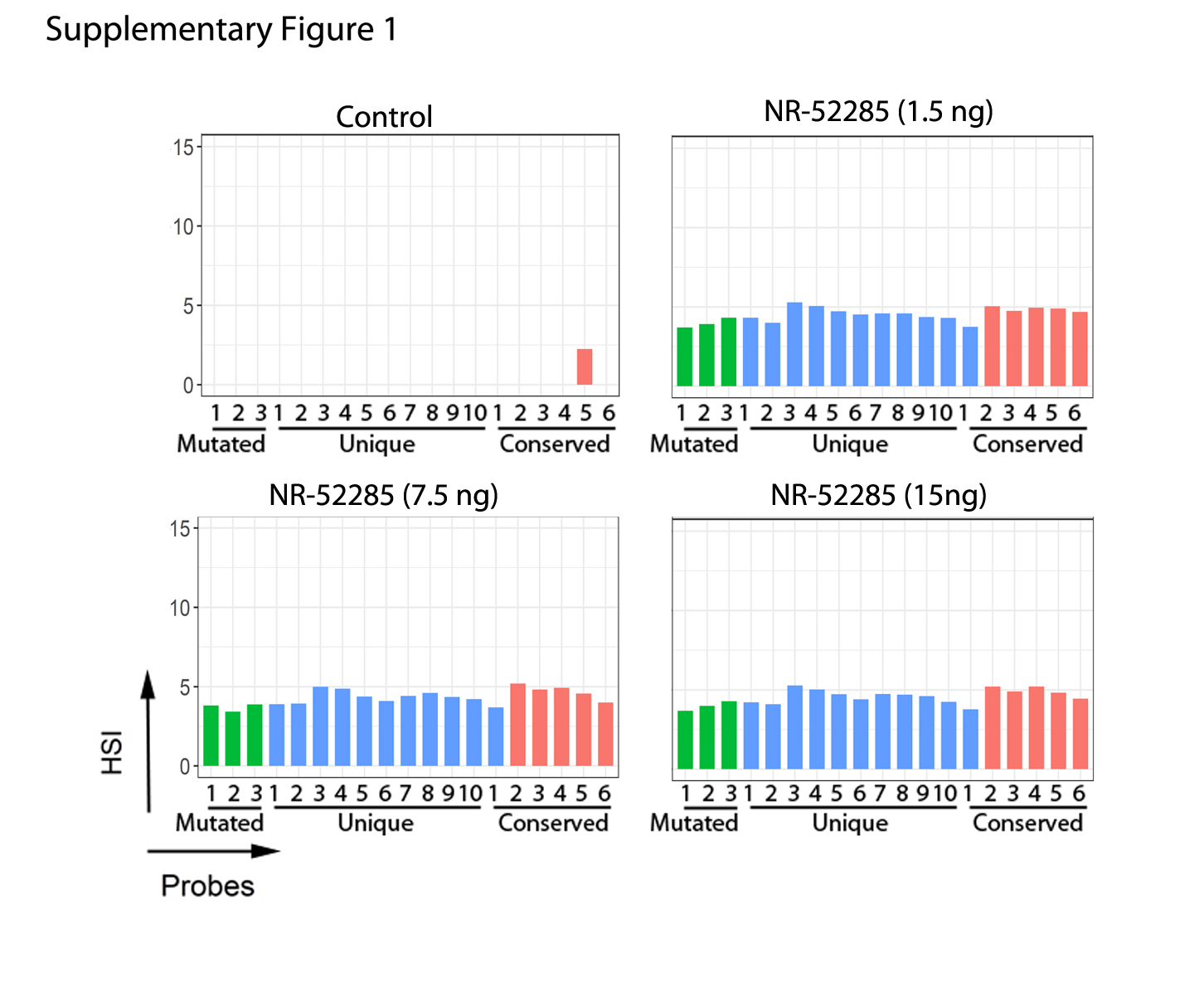

Supplement: Supplementary file 1 [file Table_1.DOCX]
